# comparisation of ABO blood groups between female patiens diagnosed with depressive disorders an bipolar affective disorders

**DOI:** 10.1192/j.eurpsy.2022.2206

**Published:** 2022-09-01

**Authors:** S. Vuk Pisk, K. Matic, E. Ivezic, N. Geres, I. Filipcic

**Affiliations:** 1 Clinical Psychiatric hospital Sveti Ivan, Department Of Integrative Psychiatry, Zagreb, Croatia; 2 Clinical Psychiatric hospital Sveti Ivan, Acute Womens Ward, Zagreb, Croatia; 3 Psychiatric Clinic “Sveti Ivan”, Psychiatry, Zagreb, Croatia

**Keywords:** ABO blood group, depressive disorder, bipolar affective dissorder, female

## Abstract

**Introduction:**

The prevalence of ABO alleles in population is different. Many studies confirmed the correlation between the occurrences of some diseases with different genotypes of ABO blood groups. Studies had shown possible differencese between patients with depressive dissorder and bipolar affective disorders according to ABO blood groups. There are contradictory results; some studies had shown significant association between blood group O and BAP, other showed relationship between unipolar depression and blood type O. Others shoedn association between involuntary depression and blood group A and negative association between blood group A and BAP.

**Objectives:**

The purpose of this study was to reassess the potential diferences between patients with depressive dissorder and bipolar affective disorders according to ABO blood groups.

**Methods:**

A total of 97 adult female psychiatric inpatients participated in this study. 57,7% were diagnosed with depressive disorder and 42,3% were diagnosed with bipolar affective disorder. Type of ABO group were measured from the blood samples taken in the morning after 30 min rest. From whole blood, genomic DNA was isolated on QIAcube device (Qiagen, Germany) using QIAamp DNA Blood mini QIAcube kit (Qiagen, Germany). ABO genotyping on 5 basic ABO alleles was performed using allele-specific PCR.

**Results:**

Comparing ABO blood groups between female patients who are suffering from depressive disorders and bipolar affective disorders, we didn’t found any differences. In both examination groups, higher proportion of A blood group was significant.

**Conclusions:**

The results of this study didn’t support the hypothesis of diferences in ABO blood group between depressive disorders and bipolar affective disorders.

**Disclosure:**

No significant relationships.

